# Pooled Platelet-Rich Plasma Lysate Therapy Increases Synoviocyte Proliferation and Hyaluronic Acid Production While Protecting Chondrocytes From Synoviocyte-Derived Inflammatory Mediators

**DOI:** 10.3389/fvets.2018.00150

**Published:** 2018-07-04

**Authors:** Jessica M. Gilbertie, Julie M. Long, Alicia G. Schubert, Alix K. Berglund, Thomas P. Schaer, Lauren V. Schnabel

**Affiliations:** ^1^Department of Clinical Sciences, College of Veterinary Medicine, North Carolina State University, Raleigh, NC, United States; ^2^Comparative Medicine Institute, North Carolina State University, Raleigh, NC, Unites States; ^3^Department of Clinical Studies New Bolton Center, School of Veterinary Medicine, University of Pennsylvania, Kennett Square, PA, United States

**Keywords:** platelet-rich plasma lysate, osteoarthritis, IL-1β, LPS, HA, collagen type II, aggrecan, MMP-13

## Abstract

Platelet-rich plasma (PRP) preparations are being used with moderate success to treat osteoarthritis (OA) in humans and in veterinary species. Such preparations are hindered, however, by being autologous in nature and subject to tremendous patient and processing variability. For this reason, there has been increasing interest in the use of platelet lysate preparations instead of traditional PRP. Platelet lysate preparations are acellular, thereby reducing concerns over immunogenicity, and contain high concentrations of growth factors and cytokines. In addition, platelet lysate preparations can be stored frozen for readily available use. The purpose of this study was to evaluate the effects of a pooled allogeneic platelet-rich plasma lysate (PRP-L) preparation on equine synoviocytes and chondrocytes challenged with inflammatory mediators *in-vitro* to mimic the OA joint environment. Our hypothesis was that PRP-L treatment of inflamed synoviocytes would protect chondrocytes challenged with synoviocyte conditioned media by reducing synoviocyte pro-inflammatory cytokine production while increasing synoviocyte anti-inflammatory cytokine production. Synoviocytes were stimulated with either interleukin-1β (IL-1β) or lipopolysaccharide (LPS) for 24 h followed by no treatment or treatment with platelet-poor plasma lysate (PPP-L) or PRP-L for 48 h. Synoviocyte growth was evaluated at the end of the treatment period and synoviocyte conditioned media was assessed for concentrations of hyaluronic acid (HA), IL-1β, tumor necrosis factor alpha (TNF-α), and interleukin-6 (IL-6). Chondrocytes were then challenged for 48 h with synoviocyte conditioned media from each stimulation and treatment group and examined for gene expression of collagen types I (COL1A1), II (COL2A1), and III (COL3A1), aggrecan (ACAN), lubricin (PRG4), and matrix metallopeptidase 3 (MMP-3) and 13 (MMP-13). Treatment of inflamed synoviocytes with PRP-L resulted in increased synoviocyte growth and increased synoviocyte HA and IL-6 production. Challenge of chondrocytes with conditioned media from PRP-L treated synoviocytes resulted in increased collagen type II and aggrecan gene expression as well as decreased MMP-13 gene expression. The results of this study support continued investigation into the use of pooled PRP-L for the treatment of osteoarthritis and warrant further *in-vitro* studies to discern the mechanisms of action of PRP-L.

## Introduction

Intra-articular injections of autologous platelet-rich plasma (PRP) are commonly used to treat osteoarthritis (OA) in humans and veterinary species, including horses and dogs (1–16). There is tremendous variability, however, in the composition of PRP generated based on the systemic health and hydration status, sex, and age of the patient, the quality of the venipuncture technique, the system or processing methods used, and whether or not the PRP is activated prior to injection (4, 17–21). The end result of such variability is large differences in platelet concentration, and therefore, growth factor and cytokine concentrations as well as leukocyte concentration within the products being used (4, 17–21). While classification systems have been put in place to define leukocyte-poor and leukocyte-rich PRPs (22–25), there is still controversy over which preparation is most efficacious for the treatment of OA and other musculoskeletal diseases (1, 4, 26–30). In addition, the optimal concentration of platelets within these preparations has yet to be elucidated (4, 26).

There has been increasing interest in the use of platelet lysate (PL) instead of PRP as PL is an acellular preparation containing high concentrations of growth factors and cytokines (31–48). The acellular nature of PL is important because it has the potential to be used in an allogeneic manner with further processing to remove immunoglobulins and also because it can be quality tested and then stored frozen to have available for immediate patient use (31–48). Furthermore, the use of pooled PL, or PL generated from multiple healthy donors, is being explored to capitalize on the natural variability that exists between individuals and the growth factors and cytokines that are released from their platelets upon lysis. This concept of optimal pooled PL has been investigated both for the use of PL as a non-immunogenic serum substitute for cell culture as well as for the use of PL as a clinical treatment (34, 41, 49).

A recent study evaluating the ability of equine PL preparations to modulate the innate immune responses of equine monocytes found interesting results when comparing data obtained from six individual PL preparations to data obtained from a pooled PL preparation created from those same six PL preparations (41). Notably, while none of the six individual PL preparations lead to significantly reduced tumor necrosis alpha (TNF-α) production from monocytes compared to fetal bovine serum (FBS), the pooled PL preparation did. Similarly, the pooled PL preparation dramatically reduced the variability observed in individual PL preparations for monocyte production of interleukin-1β (IL-1β) and interleukin-10 (IL-10). Lastly, the pooled PL preparation in this study was found to significantly decrease the production of both TNF-α and IL-1β by lipopolysaccharide (LPS) stimulated monocytes compared to controls (41). These results suggest that pooled PL preparations reduce variability and increase efficacy compared to individual PL preparations and that pooled PL preparations should be further examined as a means to suppress inflammation (41).

The aim of this study was to examine the effects of a pooled allogeneic platelet-rich plasma lysate (PRP-L) preparation on equine synoviocytes and chondrocytes challenged with inflammatory mediators *in-vitro* to mimic the OA joint environment. Our hypothesis was that PRP-L treatment of inflamed synoviocytes would protect chondrocytes challenged with synoviocyte conditioned media by reducing synoviocyte pro-inflammatory cytokine production and increasing synoviocyte anti-inflammatory cytokine production. In particular, we expected chondrocytes challenged with conditioned media from IL-1β or LPS stimulated synoviocytes treated with PRP-L to have increased gene expression of collagen type II and decreased gene expression of MMP-3 and MMP-13 compared to conditioned media from non-treated or platelet-poor plasma lysate (PPP-L) treated synoviocytes.

## Materials and methods

### Study design

**Figure 1:**
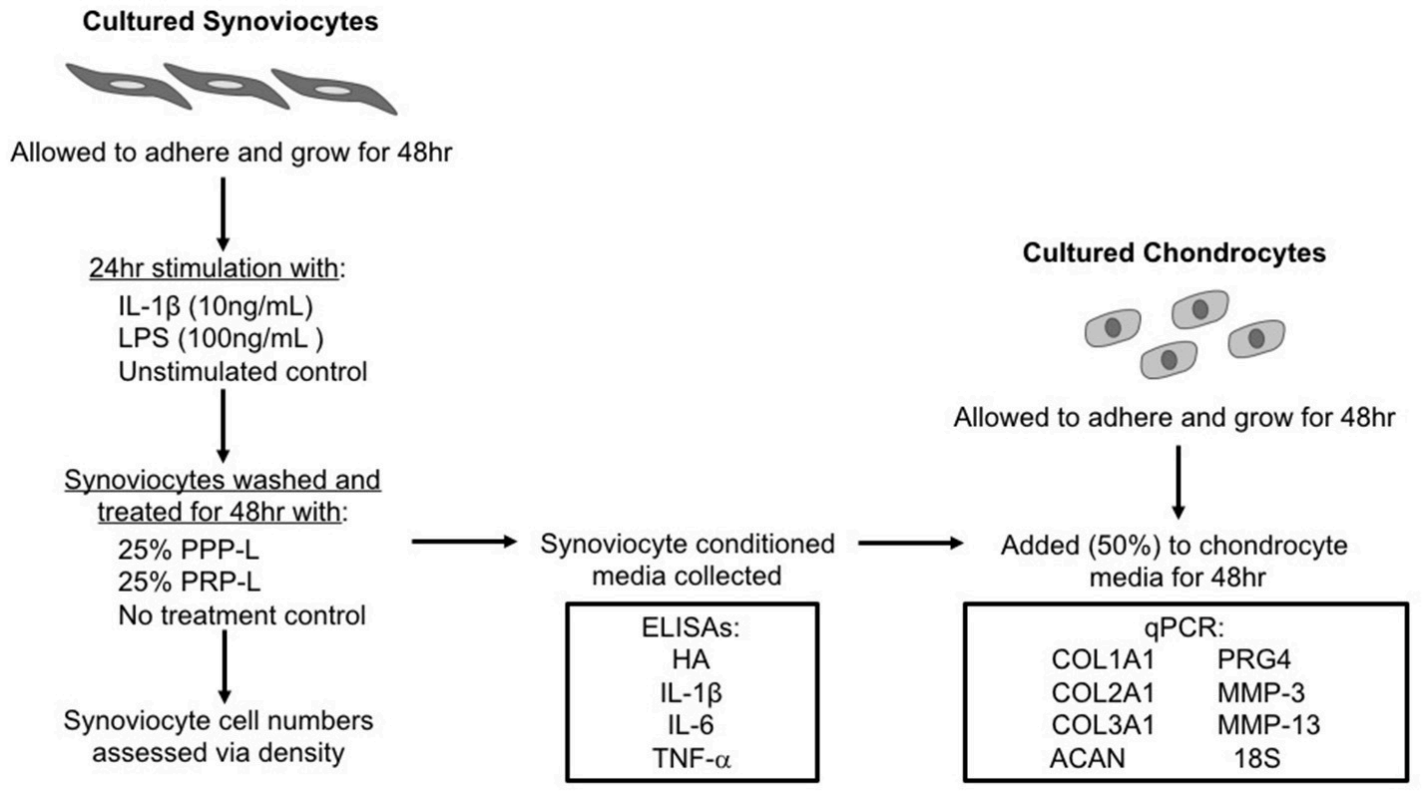
Schematic of the study design.

A schematic of the study design is shown in Figure 1. The Institutional Animal Care and Use Committee of North Carolina State University approved the use of horses in these studies.

### Platelet-poor plasma lysate and platelet-rich plasma lysate preparation

Whole blood was collected via jugular venipuncture from 6 healthy horses in our closed research herd into four 60 mL syringes containing 6 mL of acid citrate dextrose (ACD) each for a total volume of 240 mL per horse. These horses included 3 geldings and 3 nonparous mares between the ages of 6 and 19 years. Routine automated complete blood counts and platelet counts were performed on each whole blood sample. Erythrocytes were allowed to settle for 30 min in the syringes and the layer above the erythrocytes containing the leukocytes, platelets, and plasma (approximately 120 mL) was then transferred to a 50 mL conical tube and centrifuged at 250 g for 15 min. From each conical, the supernatant above the leukocyte pellet containing the platelets and plasma was then harvested and centrifuged at 1,500 g for 15 min. From this spin, the supernatant containing the platelet-poor plasma (PPP) was removed and saved. The platelet pellet was then resuspended in 12 mL of PPP to generate platelet-rich plasma (PRP) of approximately 10x the concentration of whole blood. Platelet numbers in PPP and PRP samples were determined by staining platelets with 1 μM Calcein-AM (Invitroge^TM^ Molecular Probes^TM^, ThermoFisher Scientific, Waltham, MA, USA), incubating for 20 min, and then counting the number of fluorescent cells using a Cellometer® Auto 2000 (Nexcelom Bioscience LLC, Lawrence, MA, USA). White blood cell (WBC) counts in PPP and PRP samples were determined using a Cellometer® Auto 2000 and ViaStain™ AOPI Staining Solution (Nexcelom Bioscience LLC, Lawrence, MA, USA). To generate PPP and PRP lysate, (PPP-L and PRP-L, respectively), the PPP and PRP then underwent five freeze/thaw cycles in liquid nitrogen. The majority of cell debris was removed from all PPP-L and PRP-L samples by centrifugation at 20,000 g for 20 min. PPP-L and PRP-L samples were then clarified by depth filtration using the ZetaPlus^TM^ BC25 Capsule Filter, Medi 90ZB (3M Purification Inc., St Paul, MN, USA). The resultant PPP-L and PRP-L samples were then pooled, respectively, from all 6 horses and frozen at −80°C until use.

### Synoviocyte isolation

Synovium was harvested from the femoropatellar joints of 5 systemically healthy horses (ages 2–14 years) euthanized for reasons other than this study and free of femoropatellar joint disease. The isolated synovium was weighed and digested for 2 h at 37°C under constant rotation with synoviocyte media [high glucose (4.5 g/L) DMEM medium with 10% fetal bovine serum (FBS), 2 mM l-glutamine, 1 mM sodium pyruvate, 25 mM HEPES, penicillin (100 units/mL), and streptomycin (100 μg/ml)] added at 10 mL/g tissue and containing 1.5 mg/mL Gibco® collagenase type II (ThermoFisher Scientific, Waltham, MA, USA) (50, 51). The resulting digest was passed through a 100 μm filter and centrifuged at 800 g for 10 min. The cell pellet was then washed twice with fresh synoviocyte media and live synoviocyte count was determined using a Cellometer® Auto 2000 and ViaStain^TM^ AOPI Staining Solution (Nexcelom Bioscience LLC, Lawrence, MA, USA). Synoviocytes were frozen in aliquots of 10 × 10^6^ cells/mL in liquid nitrogen until use.

### Chondrocyte isolation

Cartilage was harvested from the femoral trochlear ridges of a 2-year-old Thoroughbred gelding free of orthopedic disease and euthanized for reasons other than this study. The isolated cartilage was weighed and digested overnight (16–18 h) at 37°C under constant rotation with chondrocyte media [Ham's F12 medium with 10% FBS, 25 mM HEPES, ascorbic acid (50 μg/mL), α-ketoglutarate (30 μg/mL), L-glutamine (300 μg/mL), penicillin (100 units/mL), and streptomycin (100 μg/ml)] containing 0.75 mg/mL of Gibco® collagenase type II (ThermoFisher Scientific, Waltham, MA, USA) (52, 53). The resulting digest was passed through a 100 μm filter and centrifuged at 800 g for 10 min. The cell pellet was then washed twice with fresh chondrocyte media. Cells were resuspended in chondrocyte media and live chondrocyte count was determined using a Cellometer® Auto 2000 and ViaStain^TM^ AOPI Staining Solution. Chondrocytes were frozen in aliquots of 10 × 10^6^ cells/mL in liquid nitrogen until use.

### Synoviocyte stimulation and treatment

Cryopreserved synoviocytes were thawed, seeded onto a 12-well plate at 4 × 10^5^ cells/well in synoviocyte media (50), and maintained at 5%CO_2_, 90% humidity, and 37°C. Cells were brought to confluency over 48 h before media exchange and stimulated with either recombinant human IL-1β at 10 ng/mL (R&D Systems, Minneapolis, MN, USA) (54) or *E. Coli* O55:B5 LPS at 100 ng/mL (Sigma-Aldrich, St. Louis, MO, USA) (55, 56). Unstimulated control wells underwent media exchange only. After 24 h of stimulation, the stimulation media was removed and the cellular monolayer was washed twice with phosphate buffered saline (PBS). Fresh media was then added to the cells either alone (no treatment controls) or with 25% PPP-L or 25% PRP-L. Synoviocytes were treated for 48 h and the resultant synoviocyte conditioned media was collected, centrifuged at 2,000 g for 15 min, and divided into aliquots for cytokine analyses and chondrocyte treatments. Aliquots for cytokine analyses were frozen at −80°C until use while aliquots for chondrocyte treatments were used fresh.

### Synoviocyte quantification

After removal of the synoviocyte conditioned media as described above, 1 mL of PBS was added to each synoviocyte well. Spectrophotometric quantification of synoviocyte cell numbers in each monolayer was then measured by density using a multiple detection plate reader set at an absorbance of 800 nm (Synergy™ 2, BioTek Instruments Inc., Winooski, VT, USA) (57). Fold change in cell density was determined as a change from the unstimulated, non-treated synoviocytes.

### Synoviocyte conditioned media analyses

Hyaluronic acid (hyaluronan; HA) concentrations were quantified in duplicate aliquots of all synoviocyte conditioned media samples using the commercially available Hyaluronan Quantikine ELISA kit (R&D Systems Minneapolis, MN, USA). Standards in each kit were used to generate standard curves and samples were analyzed for optical density on a multiple detection plate reader (Synergy™ 2, BioTek Instruments Inc., Winooski VT) at 450 nm with wavelength correction set at 540 nm.

Pro-inflammatory IL-1β and TNF-α and pro/anti-inflammatory IL-6 concentrations in synoviocyte conditioned media were determined using a truncated version of the commercially available equine multiplex assay (MILLIPLEX MAP Equine Cytokine/Chemokine Magnetic Bead Panel, EMD Millipore, Burlington, MA, USA) on a MAGPIX® System (Luminex Corp., Austin, TX, USA). All samples were analyzed in duplicate using a 96-well platform performed per manufacturer's instructions. A minimum bead count of 50 for each cytokine was acquired for data analysis. Data were analyzed using Milliplex Analyst 5.1 software (Luminex Corporation, Austin, TX, USA).

### Chondrocyte challenge with synoviocyte conditioned media

Cryopreserved chondrocytes were thawed, seeded onto a 24-well plate at 2 × 10^5^ cells/well in chondrocyte media (53) and maintained at 5%CO_2_, 90% humidity, and 37°C. Cells were brought to confluency over 48 h before media exchange with an equal volume of synoviocyte conditioned media to chondrocyte media for each well. Challenge experiments were carried out for 48 h.

### Chondrocyte RNA extraction and qPCR

Total cellular RNA was extracted from chondrocytes using the RNeasy Mini Kit (Qiagen Inc., Germantown, MD, USA) according to the manufacturer's instructions. The RNA purity and quantity were evaluated using UV microspectrophotometry (NanoDrop 2000 Spectrophptometer, ThermoFisher Scientific, Waltham, MA, USA). RNA was stored at −80°C until cDNA construction by RT-PCR using the QuantiTect Reverse Transciption Kit (Qiagen Inc., Germantown, MD, USA) according to the manufacturer's instructions.

Previously published equine primers were used to amplify collagen types I (COL1A1), II (COL2A1), and III (COL3A1), aggrecan (ACAN), lubricin (PRG4), and matrix metallopeptidase 3 (MMP-3) and 13 (MMP-13) with 18S used as a housekeeping gene (Table 1). Quantitative real time RT-PCR (qPCR) was performed using the QuantiFast®SYBR®Green PCR Kit (Qiagen Inc., Germantown, MD, USA) according to the manufacturer's instructions with the QuantStudio®6 Flex System (applied biosystems®, ThermoFisher Scientific, Waltham, MA, USA). Relative gene expression, 2^−ΔΔCt^, was generated using Real-Time PCR Software v1.2 (applied biosystems®, ThermoFisher Scientific, Waltham, MA, USA). Chondrocytes cultured with the unstimulated, non-treated synoviocyte conditioned media were used as controls.

**Table 1:** Equine primer sequences used for gene expression analyses.

**Gene**	**Primer sequences**
COL1A1, Collagen type I (58)	Forward, 5′-AAGGACAAGAGGCACGTCTG-3′ Reverse, 5′-GCAGGAAAGTCAGCTGGATG−3′
COL2A1, Collagen type II (53)	Forward, 5′-GCTACACTCAAGTCCCTCAAC-3′ Reverse, 5′-ATCCAGTAGTCTCCGCTCTT-3′
COL3A1, Collagen type III (58)	Forward, 5′-GGGTATAGCTGGTCCTCGTG-3′ Reverse, 5′-GCGCCTCTTTCTCCTTTAGC−3′
ACAN, Aggrecan (59)	Forward, 5′-CAACAACAATGCCCAAGACTAC-3′ Reverse, 5′-AGTTCTCAAATTGCAAGGAGTG−3′
PRG4, Proteoglycan 4 (Lubricin) (60)	Forward, 5′-TGCGGTGCTTCCCCATAC-3′ Reverse, 5′-AAACAGGAACCCATCAGAAAGTG−3′
MMP-3, Matrix metallopeptidase 3 (53)	Forward, 5′-ATGGACCTTCTTCAGGACTACC-3′ Reverse, 5′-GACCGACATCAGGAACTCCG-3′
MMP-13, Matrix metallopeptidase 13 (53)	Forward, 5′-ACAAGCAGTTCCAAAGGCTAC-3′ Reverse, 5′-CTCGAAGACTGGTGATGGCA-3′
18S, 18 small ribonucleic acid (53)	Forward, 5′-GCCGCTAGAGGTGAAATTCT-3′ Reverse, 5′-TCGGAACTACGACGGTATCT-3′

### Statistical analyses

All results were assessed for normality by means of Shapiro-Wilk test. Normally distributed data was analyzed by the analysis of covariance (ANCOVA) with horse as covariate, followed by the Tukey's test for multiple comparisons. Non-normally distributed data was analyzed by the non-parametric Wilcoxon rank sum test. Statistical analyses were performed within the non-treated group across stimulations to assess the effects of stimulation and then within each stimulation group to assess for treatment effects. Analyses were performed using JMP® Pro11 (SAS Institute Inc., Cary, NC, USA) and significance set at *p* < 0.05. All graphs were generated with GraphPad Prism 7 (GraphPad, La Jolla, CA, USA).

## Results

### Verification of platelet-poor plasma and platelet-rich plasma lysate preparations

White blood cell (WBC) and platelet counts verified the generation of leukocyte-reduced PPP and PRP. The mean ± standard deviation (*n* = 6) WBC count in whole blood was 5.60 × 10^3^/μL ± 0.62 × 10^3^/μL compared to 0.05 × 10^3^/μL ± 0.02 × 10^3^/μL in PPP and 1.39 × 10^3^/μL ± 0.26 × 10^3^/μL in PRP. The mean ± standard deviation (*n* = 6) platelet count in whole blood was 134.50 × 10^3^/μL ± 35.67 × 10^3^/μL compared to 9.38 × 10^3^/μL ± 3.46 × 10^3^/μL in PPP and 1226.38 × 10^3^/μL ± 55.32 × 10^3^/μL in PRP. As such, the platelet concentration in each PRP sample was very close to our target of 10x the platelet concentration of whole blood.

### Effects of IL-1β or LPS stimulation on synoviocytes and chondrocytes challenged with synoviocyte conditioned media

**Figure 2:**
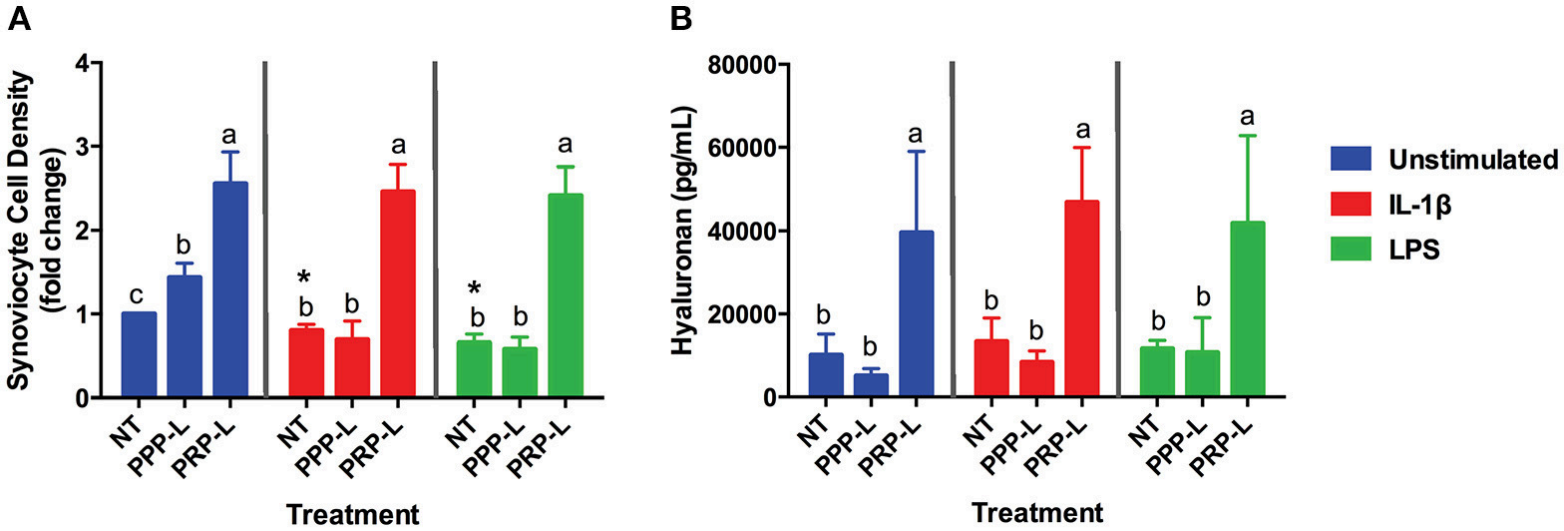
PRP-L but not PPP-L treatment increases synoviocyte growth and production of hyaluronic acid regardless of synoviocyte stimulation. Equine synoviocytes were left unstimulated or stimulated with 10 ng/mL of IL-1β or 100 ng/mL of LPS for 24 h before being non-treated (NT) or treated with platelet-poor plasma lysate (PPP-L) or platelet-rich plasma lysate (PRP-L) for 48 h. **(A)** Synoviocyte growth was measured via optical density and displayed as a fold change from the unstimulated, non-treated group. **(B)** Production of hyaluronic acid (hyaluronan; HA) was measured in the media using a commercial ELISA kit. Data is shown as the mean ± standard deviation of *n* = 5. Differing letters indicate significant differences between groups (*p* < 0.05); statistical analysis was performed within each stimulation and not between stimulation groups. Asterisks (^*^) denote significant differences, when present, in stimulated NT groups from the unstimulated NT control. Blue bars = unstimulated synoviocytes, red bars = 10 ng/mL IL-1β stimulated synoviocytes, and green bars = 100 ng/mL LPS stimulated synoviocytes.

**Figure 3:**
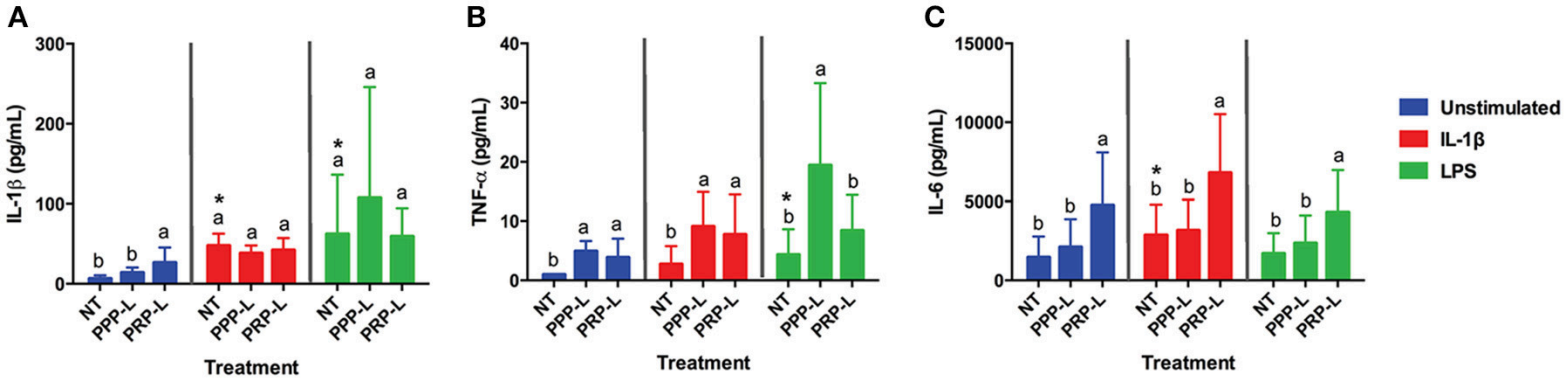
PRP-L treated synoviocytes produce more anti-inflammatory IL-6 under all stimulations conditions and less pro-inflammatory TNF-α in response to LPS stimulation compared to PPP-L treated synoviocytes. Conditioned media from cultured equine synoviocytes was collected after stimulation with 10 ng/mL of IL-1β or 100 ng/mL of LPS for 24 h and subsequent non-treatment (NT) or treatment with platelet-poor plasma lysate (PPP-L) or platelet-rich plasma lysate (PRP-L) for 48 h. Media concentrations (pg/mL) of **(A)** IL-1β, **(B)** TNF-α, and **(C)** IL-6 were measured using a commercial equine multiplex assay. Data is shown as the mean ± standard deviation of *n* = 5. Differing letters indicate significant differences between groups (*p* < 0.05); statistical analysis was performed within each stimulation and not between stimulation groups. Asterisks (^*^) denote significant differences, when present, in stimulated NT groups from the unstimulated NT control. Blue bars = unstimulated synoviocytes, red bars = 10 ng/mL IL-1β stimulated synoviocytes, and green bars = 100 ng/mL LPS stimulated synoviocytes.

Decreased synoviocyte growth compared to unstimulated controls was observed following 24 h of stimulation with either IL-1β (*p* < 0.002) or LPS (*p* < 0.001; Figure 2A). Synoviocytes stimulated with either IL-1β (*p* < 0.03) or LPS (*p* < 0.03) also had increased production of IL-1β compared to unstimulated synoviocytes (Figure 3A). Increased production of TNF-α, however, was only observed following stimulation with LPS (*p* < 0.02; Figure 3B), and increased production of IL-6 was only observed following stimulation with IL-1β (*p* < 0.03; Figure 3C). Chondrocytes challenged with synoviocyte conditioned media stimulated with either IL-1β (*p* < 0.03) or LPS (*p* < 0.03) had increased gene expression of MMP-13 (Figure 6B), while only those challenged with synoviocyte conditioned media stimulated with LPS (*p* < 0.03) had increased gene expression of MMP-3 (Figure 6A) compared to control chondrocytes challenged with unstimulated synoviocyte conditioned media.

### PRP-L increases growth and hyaluronic acid production in naïve and inflamed synoviocytes

Both PRP-L and PPP-L treatment increased the growth of unstimulated synoviocytes (*p* < 0.0003 and *p* < 0.05, respectively), but only PRP-L treatment was able to rescue the growth of synoviocytes stimulated with either IL-1β (*p* < 0.0001) or LPS (*p* < 0.0001; Figure 2A). Furthermore, only PRP-L treatment was able to increase total synoviocyte HA production in unstimulated synoviocytes (*p* < 0.005), synoviocytes stimulated with IL-1β (*p* < 0.0001) and synoviocytes stimulated with LPS (*p* < 0.009; Figure 2B). These results indicate that PRP-L treatment has powerful proliferative effects and is able to increase total synoviocyte HA production both under naïve conditions and in the face of inflammatory stimulation.

### PRP-L increases anti-inflammatory IL-6 production in naïve and inflamed synoviocytes

PRP-L treatment increased the production of IL-1β from unstimulated synoviocytes (*p* < 0.05) but did not cause any further increase in IL-1β following stimulation either IL-1β or LPS compared to non-treated synoviocytes (Figure 3A). Similarly, both PRP-L and PPP-L treatments increased the production of TNF-α from unstimulated synoviocytes (*p* < 0.05) compared to non-treated synoviocytes (Figure 3B), but neither caused any further increase in TNF-α following stimulation with IL-1β and only PPP-L treatment caused a further increase in TNF-α following LPS stimulation (*p* < 0.02). Only PRP-L treatment was able to further increase production of IL-6 from synoviocytes under all conditions compared to non-treated synoviocytes (*p* < 0.005) and compared to PPP-L treated synoviocytes (*p* < 0.05; Figure 3C). These results indicate that PRP-L treatment does not reduce pro-inflammatory cytokine production from stimulated synoviocytes but does increase anti-inflammatory IL-6 production from both unstimulated and stimulated synoviocytes compared to both non-treated and PPP-L treated synoviocytes.

### Conditioned media from PRP-L treated synoviocytes increases anabolic gene expression in cultured chondrocytes

**Figure 4:**
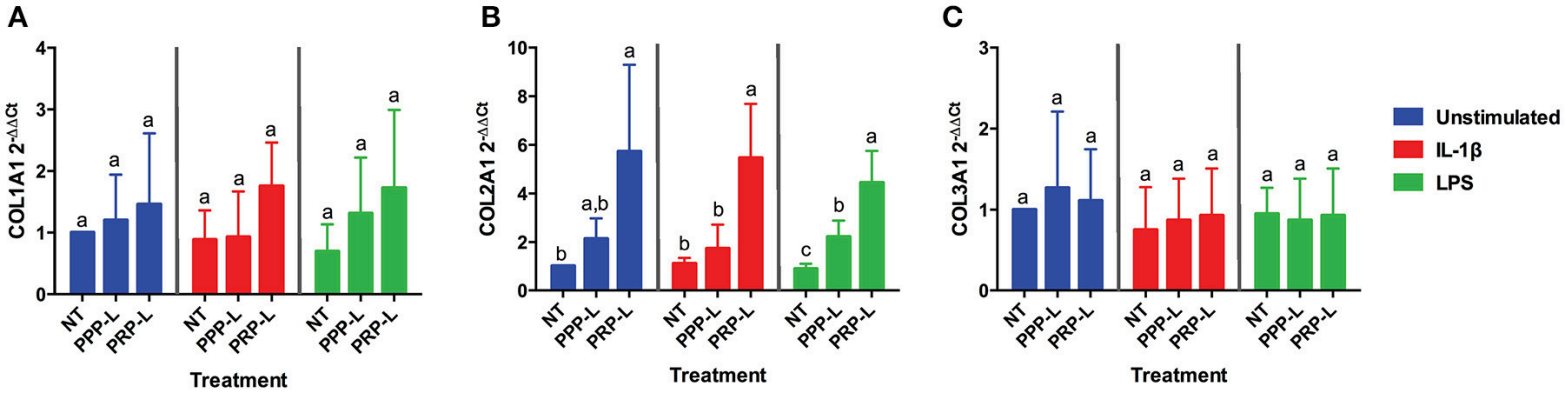
Conditioned media from synoviocytes treated with PRP-L under all stimulation conditions increases collagen type II but not collagen type I or type I in cultured chondrocytes. Equine chondrocytes were challenged for 48 h with conditioned media from synoviocytes stimulated with IL-1β or LPS and either non-treated (NT) or treated with platelet-poor plasma lysate (PPP-L) or platelet-rich plasma lysate (PRP-L). Relative gene expression is represented as the 2^−ΔΔCt^ of **(A)** collagen type I (COL1A1), **(B)** collagen type II (COL2A1), and **(C)** collagen type III (COL3A1). Data is shown as the mean ± standard deviation of n = 5. Differing letters indicate significant differences between groups (*p* < 0.05); statistical analysis was performed within each stimulation and not between stimulation groups. Asterisks (^*^) denote significant differences, when present, in stimulated NT groups from the unstimulated NT control. Blue bars = unstimulated synoviocytes, red bars = 10 ng/mL IL-1β stimulated synoviocytes, and green bars = 100 ng/mL LPS stimulated synoviocytes.

**Figure 5:**
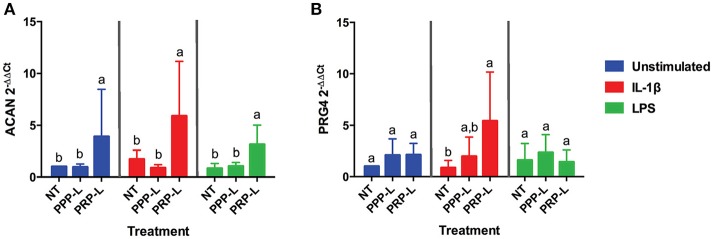
Conditioned media from synoviocytes treated with PRP-L under all stimulation conditions increases aggrecan expression in cultured chondrocytes. Equine chondrocytes were challenged for 48 h with conditioned media from synoviocytes stimulated with IL-1β or LPS and either non-treated (NT) or treated with platelet-poor plasma lysate (PPP-L) or platelet-rich plasma lysate (PRP-L). Relative gene expression represented at the 2^−ΔΔCt^ of **(A)** aggrecan (ACAN), and **(B)** lubricin (PRG4) was measured in cDNA made from extracted chondrocyte RNA. Fold changes were generated from the chondrocytes cultured with unstimulated, non-treated synoviocyte conditioned media. Data is shown as the mean ± standard deviation of *n* = 5. Differing letters indicate significant differences between groups (*p* < 0.05); statistical analysis was performed within each stimulation and not between stimulation groups. Asterisks (^*^) denote significant differences, when present, in stimulated NT groups from the unstimulated NT control. Blue bars = unstimulated synoviocytes, red bars = 10 ng/mL IL-1β stimulated synoviocytes, and green bars = 100 ng/mL LPS stimulated synoviocytes.

The anabolic effects of PPP-L treated, PRP-L treated, or non-treated synoviocytes either unstimulated or stimulated with IL-1β or LPS on cultured chondrocytes were assessed by measuring relative chondrocyte gene expression of collagen type I (Figure 4A), collagen type II (Figure 4B), collagen type III (Figure 4C), aggrecan (Figure 5A), and lubricin (Figure 5B). Chondrocytes challenged with conditioned media from PRP-L treated synoviocytes had increased collagen type II expression compared to chondrocytes challenged with conditioned media from non-treated synoviocytes when the synoviocytes where unstimulated (*p* < 0.03), IL-1β stimulated (*p* < 0.003), or LPS stimulated (*p* < 0.001) and compared to chondrocytes challenged with conditioned media from PPP-L treated synoviocytes when the synoviocytes where either IL-1β stimulated (*p* < 0.01) or LPS stimulated (*p* < 0.01; Figure 4B). No significant differences in chondrocyte gene expression of either collagen type I and collagen type III were found for any stimulation or treatment group of synoviocyte cultured media (Figures 4A,C). Chondrocytes challenged with conditioned media from PRP-L treated synoviocytes had increased aggrecan expression compared to chondrocytes challenged with conditioned media from either non-treated or PPP-L treated synoviocytes when the synoviocytes were unstimulated (*p* < 0.02), IL-1β stimulated (*p* < 0.01), or LPS stimulated (*p* < 0.02) (Figure 5A). Lubricin (proteoglycan 4) gene expression was largely unaffected apart from increased gene expression in chondrocytes challenged with PRP-L treated, IL-1β stimulated synoviocyte conditioned media (*p* < 0.05) compared to those challenged with non-treated, IL-1β stimulated synoviocyte conditioned media (Figure 5B). These results indicate that PRP-L treatment increases the production of normal collagen type II found in mature articular cartilage rather than inferior collagen type I found in fibrocartilage or collagen type III found in cartilage undergoing repair. In addition, PRP-L treatment increases the production of aggrecan, the major structural proteoglycan of cartilage extracellular matrix.

### Conditioned media from both PRP-L and PPP-L treated inflamed synoviocytes decreases catabolic gene expression in cultured chondrocytes

**Figure 6:**
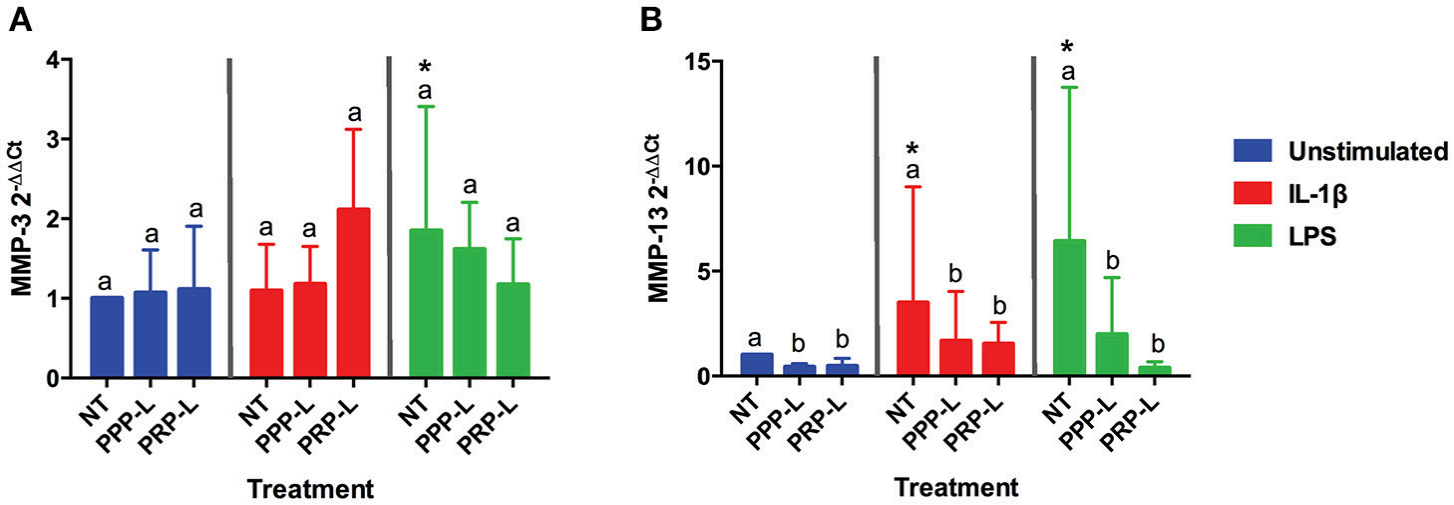
Conditioned media from synoviocytes treated with PRP-L or PPP-L under all stimulation conditions decreases MMP-13 but not MMP-3 gene expression in cultured chondrocytes. Equine chondrocytes were challenged for 48 h with conditioned media from synoviocytes stimulated with IL-1β or LPS and either non-treated (NT) or treated with platelet-poor plasma lysate (PPP-L) or platelet-rich plasma lysate (PRP-L). Relative gene expression, 2^−ΔΔCt^, was generated using the unstimulated, non-treated conditioned media cultured chondrocytes as the control for **(A)** MMP-3 and **(B)** MMP-13. Data is shown as the mean ± standard deviation of *n* = 5. Differing letters indicate significant differences between groups (*p* < 0.05); statistical analysis was performed within each stimulation and not between stimulation groups. Asterisks (^*^) denote significant differences, when present, in stimulated NT groups from the unstimulated NT control. Blue bars = unstimulated synoviocytes, red bars = 10 ng/mL IL-1β stimulated synoviocytes, and green bars = 100 ng/mL LPS stimulated synoviocytes.

The catabolic effects of PPP-L treated, PRP-L treated, or non-treated synoviocytes either unstimulated or stimulated with IL-1β or LPS on cultured chondrocytes were assessed by measuring relative chondrocyte gene expression of MMP-3 (Figure 6A) and MMP-13 (Figure 6B). Although no significant differences in chondrocyte gene expression of MMP-3 were found for conditioned media from PRP-L or PPP-L treated synoviocytes stimulated with either IL-1β or LPS compared to non-treated synoviocytes, there was a trend toward reduced MMP-3 gene expression under LPS stimulation conditions (*p* = 0.09) and in particular the PRP-L treated group decreased back down to the same level of MMP-3 gene expression observed in control chondrocytes challenged with conditioned media from unstimulated synoviocytes (Figure 6A). Chondrocytes challenged with conditioned media from both PRP-L and PPP-L treated synoviocytes had decreased MMP-13 gene expression compared to chondrocytes challenged with conditioned media from non-treated synoviocytes when the synoviocytes were unstimulated (*p* < 0.03) and also when the synoviocytes where stimulated with IL-1β (*p* < 0.05) or LPS (*p* < 0.02; Figure 6B). Similar to the trend observed for chondrocyte gene expression of MMP-3 under LPS stimulation conditions, there was a trend toward chondrocytes challenged with conditioned media from PRP-L treated synoviocytes that were stimulated with LPS to have further reduced MMP-13 gene expression compared to chondrocytes challenged with conditioned media from PPP-L treated synoviocytes that were stimulated with LPS (Figure 6B). These results indicate that both PRP-L and PPP-L treatments are able to downregulate MMP-13 production in chondrocytes under LPS stimulation conditions, but with PRP-L treatment trending toward a closer return to unstimulated control levels of MMP-13.

## Discussion

The aim of this study was to examine the effects of a pooled allogeneic platelet-rich plasma lysate (PRP-L) preparation on equine synoviocytes and chondrocytes challenged with inflammatory mediators *in-vitro* to mimic the OA joint environment. The findings of this study support our overall hypothesis that PRP-L treatment of inflamed synoviocytes protects chondrocytes challenged with synoviocyte conditioned media. The protective effect of PRP-L, however, appears to be more through increase of synoviocyte anti-inflammatory cytokine production rather than through reduction of synoviocyte pro-inflammatory mediators. Such findings are consistent with those of previous studies on the anti-inflammatory effects of PL on other cell types (35, 41, 61) and are also consistent with previous studies on the anabolic and anti-catabolic effects of PRP preparations on chondrocytes (62–65). A particularly interesting, and unexpected finding of this study, however, was that treatment with PRP-L stimulated the growth of synoviocytes and the production of HA from synoviocytes even when challenged with IL-1β or LPS.

Numerous studies evaluating the efficacy of PL as a serum replacement for FBS in cell culture media have supported the proliferative effect of PL preparations on different cell types including bone marrow-derived stromal cells, adipose-derived stromal cells, synovial fluid stromal cells, and corneal endothelium cells (31, 38–40, 42, 45, 47). It is therefore not surprising that the PRP-L used in this study would lead to enhanced cell growth of naïve synoviocytes under normal tissue culture conditions. It is surprising, though, that very similar fold changes in synoviocyte growth were observed for synoviocytes stimulated with either IL-1β or LPS and treated with PRP-L. The dramatic increases in synoviocyte growth observed under naïve and stimulated conditions when treated with PRP-L were consistent with the dramatic increases observed in HA production by synoviocytes under naïve and stimulated conditions when treated with PRP-L. PRP preparations have been previously reported to increase protein production of HA and to increase gene expression of Hyaluronan synthase-2 (HAS-2) in synoviocytes isolated from OA patients (63, 66). There is also a report of two different platelet gel supernatants isolated from a single horse that were able to increase HA production from synoviocytes under LPS stimulation compared to controls, but not to the same extent as observed in this current study (67). It is possible that the pooled nature of the PRP-L used in this study may have been in part responsible for the differences observed in HA production compared to previous studies evaluating a platelet preparation from a single donor. As discussed earlier, there is existing evidence in the literature to support increased efficacy of pooled PL preparations compared to individual donor preparations, as pooled preparations are able to capitalize on the natural cytokine variability that occurs in donors (31, 41). In this current study, we were unable to discern whether or not the dramatic increases in total HA concentration were due solely to the increases in synoviocyte cell numbers caused by PRP-L treatment or due in part to upregulation of HA production by synoviocytes treated with PRP-L. Future studies examining HAS-2 gene expression in synoviocytes following treatment with PRP-L are certainly warranted to determine all mechanisms involved.

The contribution of such a remarkable increase in synoviocyte HA production following PRP-L treatment on chondrocyte gene expression is unknown and also warrants further exploration. In our current study design, it is not possible to discern if the increases in collagen type II and aggrecan gene expression and the decrease in MMP-13 gene expression that were observed are due primarily to the high concentration of HA in the conditioned media of synoviocytes treated with PRP-L or due to other variables such as the high concentrations of platelet-derived growth factors found in PRP-L, the significant increase in synoviocyte IL-6 production caused by PRP-L treatment, or other factors that were not examined for. HA is known to bind to the cluster of differentiation 44 (CD44) receptors and thereby inhibit IL-1β expression resulting in decreased MMP production (68, 69). HA has also been shown to increase the proliferation of chondrocytes in tissue culture as well as to stimulate them to produce more collagen type II and aggrecan (70). Although IL-6 can be pro-inflammatory under certain conditions, it is known to induce the production of IL-1 and TNF antagonists by a variety of cell types including macrophages (71) and has been highlighted as a critical cytokine for the repair of other musculoskeletal tissues such as tendon (72, 73).

Another factor to consider that was not examined in this current study is the role of hypoxia-inducible factor (HIF) in PRP-L mediated chondroprotection and cartilage repair (37, 74–76). Several studies have previously demonstrated the critical role of HIF-1α in cartilage and in the nucleus pulposus for maintaining proper cellular function, including synthesis of extracellular matrix proteins, in a hypoxic environment (76–79). Both articular cartilage and the nucleus pulposus are highly avascular tissues with low intrinsic healing capacity and low oxygen tensions. This unique environment requires mechanisms adapted to support the survival of the tissue's resident cells, and HIF-1α is considered to be one of the main elements in such mechanisms. A recent study evaluating the effect of a pooled human PL preparation on growth-arrested progenitor cartilage cells found that PL induced the re-entry of such cells into the cell cycle (37). The cell activation and proliferation observed in this study was shown to correspond to induction of HIF-1 by PL (37). Consequently, we would speculate that PRP-L treatment of inflamed or damaged cartilage may activate the HIF-1 pathway to increase cell proliferation and matrix synthesis, but this remains to be investigated.

In conclusion, treatment of inflamed synoviocytes with PRP-L *in-vitro* resulted in increased synoviocyte growth and increased total synoviocyte HA and IL-6 production. Challenge of chondrocytes with conditioned media from PRP-L treated synoviocytes then resulted in increased collagen type II and aggrecan gene expression as well as decreased MMP-13 gene expression. The results of this study support continued investigation into the use of pooled PRP-L for the treatment of osteoarthritis and warrant further *in-vitro* studies to discern the mechanisms of action of PRP-L.

## Author contributions

We certify that all authors meet the qualifications for authorship as listed below: (1) substantial contributions to conception or design of the work or the acquisition, analysis, or interpretation of data for the work; (2) drafting the work or revising it critically for important intellectual content; (3) final approval of the version to be published; (4) agreement to be accountable for all aspects of the work in ensuring that questions related to the accuracy or integrity of any part of the work are appropriately investigated and resolved.

### Conflict of interest statement

The authors declare that the research was conducted in the absence of any commercial or financial relationships that could be construed as a potential conflict of interest.

## References

[1] Riboh JC Saltzman BM Yanke AB Fortier L Cole BJ. Effect of leukocyte concentration on the efficacy of platelet-rich plasma in the treatment of knee osteoarthritis. Am J Sports Med. (2016) 44:792–800. 10.1177/0363546515580787 25925602

[2] Jubert NJ Rodríguez L Reverté-Vinaixa MM Navarro A. Platelet-rich plasma injections for advanced knee osteoarthritis: a prospective, randomized, double-blinded clinical trial. Orthop J Sport Med. (2017) 5:1–11. 10.1177/2325967116689386 28255569 PMC5315239

[3] Cole BJ Karas V Hussey K Merkow DB Pilz K Fortier LA. Hyaluronic acid versus platelet-rich plasma: a prospective, double-blind randomized controlled trial comparing clinical outcomes and effects on intra-articular biology for the treatment of knee osteoarthritis. Am J Sports Med. (2017) 45:339–46. 10.1177/0363546516665809 28146403

[4] McCarrel TM Mall NA Lee AS Cole BJ Butty DC Fortier LA. Considerations for the use of platelet-rich plasma in orthopedics. Sport Med. (2014) 44:1025–36. 10.1007/s40279-014-0195-5 24760591

[5] Shen L Yuan T Chen S Xie X Zhang C. The temporal effect of platelet-rich plasma on pain and physical function in the treatment of knee osteoarthritis: systematic review and meta-analysis of randomized controlled trials. J Orthop Surg Res. (2017) 12:1–12. 10.1186/s13018-017-0521-3 28115016 PMC5260061

[6] Filardo G Kon E Buda R Timoncini A Di Martino A Cenacchi A et al. Platelet-rich plasma intra-articular knee injections for the treatment of degenerative cartilage lesions and osteoarthritis. Knee Surg Sport Traumatol Arthrosc. (2011) 19:528–35. 10.1007/s00167-010-1238-6 20740273

[7] Kon E Mandelbaum B Buda R Filardo G Delcogliano M Timoncini A et al. Platelet-rich plasma intra-articular injection versus hyaluronic acid viscosupplementation as treatments for cartilage pathology: from early degeneration to osteoarthritis. Arthrosc J Arthrosc Relat Surg. (2011) 27:1490–501. 10.1016/j.arthro.2011.05.011 21831567

[8] Filardo G Kon E Roffi A Di Matteo B Merli ML Marcacci M. Platelet-rich plasma: why intra-articular? A systematic review of preclinical studies and clinical evidence on PRP for joint degeneration. Knee Surg Sport Traumatol Arthrosc. (2015) 23:2459–74. 10.1007/s00167-013-2743-1 24275957 PMC4541701

[9] Dold AP Zywiel MG Taylor DW Dwyer T Theodoropoulos J. Platelet-rich plasma in the management of articular cartilage pathology: a systematic review. Clin J Sport Med. (2014) 24:31–43. 10.1097/01.jsm.0000432855.85143.e5 24231930

[10] Matteo BD Kon E Filardo G. Intra-articular platelet-rich plasma for the treatment of osteoarthritis. Ann Transl Med. (2016) 4:63. 10.3978/j.issn.2305-5839.2016.01.18 26904585 PMC4740013

[11] Bennell KL Hunter DJ Paterson KL. Platelet-rich plasma for the management of hip and knee osteoarthritis. Curr Rheumatol Rep. (2017) 19:24. 10.1007/s11926-017-0652-x 28386761

[12] Broeckx S Zimmerman M Crocetti S Suls M Mariën T Ferguson SJ et al. Regenerative therapies for equine degenerative joint disease: a preliminary study. PLoS ONE (2014) 9:e85917. 10.1371/journal.pone.0085917 24465787 PMC3896436

[13] Mirza MH Bommala P Richbourg HA Rademacher N Kearney MT Lopez MJ. Gait changes vary among horses with naturally occurring osteoarthritis following intra-articular administration of autologous platelet-rich plasma. Front Vet Sci. (2016) 3:29. 10.3389/fvets.2016.00029 27148544 PMC4829588

[14] Franklin SP Garner BC Cook JL. Characteristics of canine platelet-rich plasma prepared with five commercially available systems. Am J Vet Res. (2015) 76:822–7. 10.2460/ajvr.76.9.822 26309111

[15] Franklin SP Stoker AM Bozynksi CC Kuroki K Clarke KM Johnson JK et al. Comparison of platelet-rich plasma, stromal vascular fraction (SVF), or SVF with an injectable PLGA nanofiber scaffold for the treatment of osteochondral injury in dogs. J Knee Surg. (2017) [Epub ahead of print]. 10.1055/s-0037-1606575 28915522

[16] Brossi PM Moreira JJ Machado TSL Baccarin RYA. Platelet-rich plasma in orthopedic therapy: a comparative systematic review of clinical and experimental data in equine and human musculoskeletal lesions. BMC Vet Res. (2015) 11:98. 10.1186/s12917-015-0403-z 25896610 PMC4449579

[17] Boswell SG Cole BJ Sundman EA Karas V Fortier LA. Platelet-rich plasma: a milieu of bioactive factors. Arthroscopy (2012) 28:429–39. 10.1016/j.arthro.2011.10.018 22284405

[18] Hsu WK Mishra A Rodeo SR Fu F Terry MA Randelli P et al. Platelet-rich plasma in orthopaedic applications: evidence-based recommendations for treatment. J Am Acad Orthop Surg. (2013) 21:739–48. 10.5435/JAAOS-21-12-739 24292930

[19] Xiong G Lingampalli N Koltsov JCB Leung LL Bhutani N Robinson WH et al. Men and women differ in the biochemical composition of platelet-rich plasma. Am J Sports Med. (2018) 46:409–19. 10.1177/0363546517740845 29211968 PMC8487642

[20] Schippinger G Prüller F Divjak M Mahla E Fankhauser F Rackemann S et al. Autologous platelet-rich plasma preparations: influence of nonsteroidal anti-inflammatory drugs on platelet function. Orthop J Sport Med. (2015) 3:1–6. 10.1177/2325967115588896 26665098 PMC4622369

[21] Rinnovati R Romagnoli N Gentilini F Lambertini C Spadari A. The influence of environmental variables on platelet concentration in horse platelet-rich plasma. Acta Vet Scand. (2016) 58:45. 10.1186/s13028-016-0226-3 27377748 PMC4932754

[22] M Dohan Ehrenfest D, Bielecki T, Mishra A, Borzini P, Inchingolo F, et al. In search of a consensus terminology in the field of platelet concentrates for surgical use: platelet-rich plasma (PRP), platelet-rich fibrin (PRF), fibrin gel polymerization and leukocytes. Curr Pharm Biotechnol. (2012) 13:1131–7. 10.2174/138920112800624328 21740379

[23] Ehrenfest DMD Sammartino G Shibli JA Wang H Zou D Bernard J Guidelines for the publication of articles related to platelet concentrates (Platelet-Rich Plasma-PRP, or Platelet-Rich Fibrin-PRF): the international classification of the POSEIDO. POSEIDO J. (2013) 1:17–27.

[24] Ehrenfest DMD Bielecki T Corso M Del Inchingolo F Sammartino G Shedding light in the controversial terminology for platelet-rich products: platelet-rich plasma (PRP), platelet-rich fibrin (PRF), platelet-leukocyte gel (PLG), preparation rich in growth factors (PRGF), classification and commercialism. J Biomed Mater Res. (2010) 95:1280–2. 10.1002/jbm.a.32894 20925082

[25] Dohan EDM Classification of platelet concentrates (Platelet-Rich Plasma-PRP, Platelet-Rich Fibrin-PRF) for topical and infiltrative use in orthopedic and sports medicine: current consensus, clinical implications and perspectives. Muscle Ligaments Tendons J. (2014) 4:3–9. PMC4049647 24932440

[26] Boswell SG Schnabel LV Mohammed HO Sundman EA Minas T Fortier LA. Increasing platelet concentrations in leukocyte-reduced platelet-rich plasma decrease collagen gene synthesis in tendons. Am J Sports Med. (2014) 42:42–9. 10.1177/0363546513507566 24136860

[27] McCarrel TM Minas T Fortier LA. Optimization of leukocyte concentration in platelet-rich plasma for the treatment of tendinopathy. J Bone Joint Surg. (2012) 94:e143(1–8). 10.2106/JBJS.L.00019 23032594

[28] Cross JA Cole BJ Spatny KP Sundman E Romeo AA Nicholson GP et al. Leukocyte-reduced platelet-rich plasma normalizes matrix metabolism in torn human rotator cuff tendons. Am J Sports Med. (2015) 43:2898–906. 10.1177/0363546515608157 26460099

[29] Zhang L Chen S Chang P Bao N Yang C Ti Y et al. Harmful effects of leukocyte-rich platelet-rich plasma on rabbit tendon stem cells *in vitro*. Am J Sports Med. (2016) 44:1941–51. 10.1177/0363546516644718 27184544

[30] Zhou Y Zhang J Wu H Hogan MCV Wang JHC. The differential effects of leukocyte-containing and pure platelet-rich plasma (PRP) on tendon stem/progenitor cells-implications of PRP application for the clinical treatment of tendon injuries. Stem Cell Res Ther. (2015) 6:173. 10.1186/s13287-015-0172-4 26373929 PMC4572462

[31] Altaie A Use of platelet lysate for bone regeneration - are we ready for clinical translation? World J Stem Cells (2016) 8:47w 10.4252/wjsc.v8.i2.47 26981170 PMC4766250

[32] Santo VE Popa EG Mano JF Gomes ME Reis RL. Natural assembly of platelet lysate-loaded nanocarriers into enriched 3D hydrogels for cartilage regeneration. Acta Biomater. (2015) 19:56–65. 10.1016/j.actbio.2015.03.015 25795623

[33] Moreira Teixeira LS Leijten JCH Wennink JWH Chatterjea AG Feijen J van Blitterswijk CA et al The effect of platelet lysate supplementation of a dextran-based hydrogel on cartilage formation. Biomaterials (2012) 33:3651–61. 10.1016/j.biomaterials.2012.01.051 22349290

[34] Klatte-Schulz F Schmidt T Uckert M Scheffler S Kalus U Rojewski M et al. Comparative analysis of different platelet lysates and platelet rich preparations to stimulate tendon cell biology: an *in vitro* study. Int J Mol Sci. (2018) 19:1–18. 10.3390/ijms19010212 29320421 PMC5796161

[35] Pereira RC Scaranari M Benelli R Strada P Reis RL Cancedda R et al. Dual effect of platelet lysate on human articular cartilage: a maintenance of chondrogenic potential and a transient proinflammatory activity followed by an inflammation resolution. Tissue Eng Part A (2013) 19:1476–88. 10.1089/ten.tea.2012.0225 23360471

[36] Del Bue M Ricco S Conti V Merli E Ramoni R Grolli S Platelet lysate promotes in vitro proliferation of equine mesenchymal stem cells and tenocytes. Vet Res Commun. (2007) 31(Suppl. 1):289–92. 10.1007/s11259-007-0099-z 17682897

[37] Nguyen VT Cancedda R Descalzi F. Platelet lysate activates quiescent cell proliferation and reprogramming in human articular cartilage: Involvement of hypoxia inducible factor 1. J Tissue Eng Regen Med. (2017) 12:1–13. 10.1002/term.2595 29052350

[38] Altaie A Baboolal TG Wall O Jones E McGonagle D. Platelet lysate enhances synovial fluid multipotential stromal cells functions: implications for therapeutic use. Cytotherapy (2018) 20:375–84. 10.1016/j.jcyt.2017.12.003 29398623

[39] Hildner F Eder MJ Hofer K Aberl J Redl H van Griensven M et al. Human platelet lysate successfully promotes proliferation and subsequent chondrogenic differentiation of adipose-derived stem cells: a comparison with articular chondrocytes. J Tissue Eng Regen Med. (2015) 9:808–18. 10.1002/term.1649 23303715

[40] Astori G Amati E Bambi F Bernardi M Chieregato K Schäfer R et al. Platelet lysate as a substitute for animal serum for the ex-vivo expansion of mesenchymal stem/stromal cells: present and future. Stem Cell Res Ther. (2016) 7:1–8. 10.1186/s13287-016-0352-x 27411942 PMC4944312

[41] Naskou MC Norton NA Copland IB Galipeau J Peroni JF. Innate immune responses of equine monocytes cultured in equine platelet lysate. Vet Immunol Immunopathol. (2018) 195:65–71. 10.1016/j.vetimm.2017.11.005 29249319

[42] Seo J pil Tsuzuki N Haneda S Yamada K Furuoka H Tabata Y et al. Comparison of allogeneic platelet lysate and fetal bovine serum for *in vitro* expansion of equine bone marrow-derived mesenchymal stem cells. Res Vet Sci. (2013) 95:693–8. 10.1016/j.rvsc.2013.04.024 23683731

[43] Sumner SM Naskou MC Thoresen M Copland I Peroni JF. Platelet lysate obtained via plateletpheresis performed in standing and awake equine donors. Transfusion (2017) 57:1755–62. 10.1111/trf.14124 28439897

[44] Copland IB Garcia MA Waller EK Roback JD Galipeau J. The effect of platelet lysate fibrinogen on the functionality of MSCs in immunotherapy. Biomaterials (2013) 34:7840–50. 10.1016/j.biomaterials.2013.06.050 23891515

[45] Wang TJ Chen MS Chou ML Lin HC Seghatchian J Burnouf T. Comparison of three human platelet lysates used as supplements for in vitro expansion of corneal endothelium cells. Transfus Apher Sci. (2017) 56:769–73. 10.1016/j.transci.2017.08.021 28939367

[46] Tyrnenopoulou P Diakakis N Karayannopoulou M Savvas I Koliakos G. Evaluation of intra-articular injection of autologous platelet lysate (PL) in horses with osteoarthritis of the distal interphalangeal joint. Vet Q. (2016) 36:56–62. 10.1080/01652176.2016.1141257 26828234

[47] Søndergaard RH Follin B Lund LD Juhl M Ekblond A Kastrup J et al. Senescence and quiescence in adipose-derived stromal cells: Effects of human platelet lysate, fetal bovine serum and hypoxia. Cytotherapy (2017) 19:95–106. 10.1016/j.jcyt.2016.09.006 27771213

[48] Al-Ajlouni J Awidi A Samara O Al-Najar M Tarwanah E Saleh M et al. Safety and efficacy of autologous intra-articular platelet lysates in early and intermediate knee osteoarthrosis in humans:a prospective open-label study. Clin J Sport Med. (2015) 25:524–8. 10.1097/JSM.0000000000000166 25387167

[49] Burnouf T Strunk D Koh MBC Schallmoser K Human platelet lysate: replacing fetal bovine serum as a gold standard for human cell propagation? Biomaterials (2016) 76:371–87. 10.1016/j.biomaterials.2015.10.065 26561934

[50] Maher MC Schnabel LV Cross JA Papich MG Divers TJ Fortier LA. Plasma and synovial fluid concentration of doxycycline following low-dose, low-frequency administration, and resultant inhibition of matrix metalloproteinase-13 from interleukin-stimulated equine synoviocytes. Equine Vet J. (2014) 46:198–202. 10.1111/evj.12139 23855565

[51] Gregg AJ Fortier LA Mohammed HO Mayr KG Miller BJ Haupt JL Assessment of the catabolic effects of interleukin-1ß on proteoglycan metabolism in equine cartilage cocultured with synoviocytes. Am J Vet Res. (2006) 67:957–62. 10.2460/ajvr.67.6.957 16740087

[52] Nixon AJ Lust G Vernier-Singer M. Isolation, propagation, and cryopreservation of equine articular chondrocytes. Am J Vet Res. (1992) 53:2364–70. 1476323

[53] Ortved KF Austin BS Scimeca MS Nixon AJ. RNA interference mediated interleukin-1β silencing in inflamed chondrocytes decreases target and downstream catabolic responses. Arthritis (2016) 2016:3484961. 10.1155/2016/3484961 27073697 PMC4814636

[54] Byron CR Trahan RA. Comparison of the effects of interleukin-1 on equine articular cartilage explants and cocultures of osteochondral and synovial explants. Front Vet Sci. (2017) 4:152. 10.3389/fvets.2017.00152 28979900 PMC5611359

[55] Carmona JU Ríos DL López C Álvarez ME Pérez JE. Proinflammatory and anabolic gene expression effects of platelet-rich gel supernatants on equine synovial membrane explants challenged with lipopolysaccharide. Vet Med Int. (2017) 2017: 6059485. 10.1155/2017/6059485 28761774 PMC5518502

[56] Carmona JU Ríos DL López C Álvarez ME Pérez JE Bohórquez ME. *In vitro* effects of platelet-rich gel supernatants on histology and chondrocyte apoptosis scores, hyaluronan release and gene expression of equine cartilage explants challenged with lipopolysaccharide. BMC Vet Res. (2016) 12:135. 10.1186/s12917-016-0759-8 27369779 PMC4929746

[57] Mohler WA Charlton CA Blau HM. Spectrophotometric quantitation of tissue culture cell number in any medium. Biotechniques. 21:260–6. 8862811 10.2144/96212st03

[58] Bussche L Harman RM Syracuse BA Plante EL Lu Y-C Curtis TM et al. Microencapsulated equine mesenchymal stromal cells promote cutaneous wound healing *in vitro*. Stem Cell Res Ther. (2015) 6:66. 10.1186/s13287-015-0037-x 25889766 PMC4413990

[59] Pawlak E Wang L Johnson PJ Nuovo G Taye A Belknap JK et al. Distribution and processing of a disintegrin and metalloproteinase with thrombospondin motifs-4, aggrecan, versican, and hyaluronan in equine digital laminae. Am J Vet Res. (2012) 73:1035–46. 10.2460/ajvr.73.7.1035 22738056 PMC3535468

[60] Reesink HL Watts AE Mohammed HO Jay GD Nixon AJ. Lubricin/proteoglycan 4 increases in both experimental and naturally occurring equine osteoarthritis. Osteoarthr Cartil. (2017) 25:128–37. 10.1016/j.joca.2016.07.021 27498214 PMC5489058

[61] Kim H-J Yeom JS Koh Y-G Yeo J-E Kang K-T Kang Y-M et al. Anti-inflammatory effect of platelet-rich plasma on nucleus pulposus cells with response of TNF-α and IL-1. J Orthop Res. (2014) 32:551–6. 10.1002/jor.22532 24338609

[62] Akeda K An HS Okuma M Attawia M Miyamoto K Thonar EJMA et al. Platelet-rich plasma stimulates porcine articular chondrocyte proliferation and matrix biosynthesis. Osteoarthr Cartil. (2006) 14:1272–80. 10.1016/j.joca.2006.05.008 16820306

[63] Sundman EA Cole BJ Karas V Della Valle C Tetreault MW Mohammed HO et al. The anti-inflammatory and matrix restorative mechanisms of platelet-rich plasma in osteoarthritis. Am J Sports Med. (2014) 42:35–41. 10.1177/0363546513507766 24192391

[64] Simental-Mendia M Vilchez-Cavazos F Garcia-Garza R Lara-Arias J Montes-de-Oca-Luna R Said-Fernandez S et al. The matrix synthesis and anti-inflammatory effect of autologous leukocyte-poor platelet rich plasma in human cartilage explants. Histol Histopathol. (2018) 33:609–18. 10.14670/HH-11-961 29313321

[65] Van Buul GM Koevoet WLM Kops N Bos PK Verhaar JA Weinans H et al Platelet-rich plasma releasate inhibits inflammatory processes in osteoarthritic chondrocytes. Am J Sports Med. (2011) 39:2362–70. 10.1177/0363546511419278 21856929

[66] Anitua E Sánchez M Nurden AT Zalduendo MM De la Fuente M Azofra J et al. Platelet-released growth factors enhance the secretion of hyaluronic acid and induce hepatocyte growth factor production by synovial fibroblasts from arthritic patients. Rheumatology (2007) 46:1769–72. 10.1093/rheumatology/kem234 17942474

[67] Ríos DL López C Álvarez ME Samudio IJ Carmona JU Effects over time of two platelet gel supernatants on growth factor, cytokine and hyaluronan concentrations in normal synovial membrane explants challenged with lipopolysaccharide Pathophysiology of musculoskeletal disorders. BMC Musculoskelet Disord. (2015) 16:153 10.1186/s12891-015-0605-3 26092588 PMC4475292

[68] Altman RD Manjoo A Fierlinger A Niazi F Nicholls M. The mechanism of action for hyaluronic acid treatment in the osteoarthritic knee: a systematic review. BMC Musculoskelet Disord. (2015) 16:321. 10.1186/s12891-015-0775-z 26503103 PMC4621876

[69] Karna E Miltyk W Surazynski A Pałka JA. Protective effect of hyaluronic acid on interleukin-1-induced deregulation of beta1-integrin and insulin-like growth factor-I receptor signaling and collagen biosynthesis in cultured human chondrocytes. Mol Cell Biochem. (2008) 308:57–64. 10.1007/s11010-007-9612-5 17899316

[70] Ehlers EM Behrens P Wunsch L Kuhnel W Russlies M. Effects of hyaluronic acid on the morphology and proliferation of human chondrocytes in primary cell culture. Ann Anat. (2001) 183:13–7. 10.1016/S0940-9602(01)80007-8 11206979

[71] Tilg H Trehu E Atkins MB Dinarello CA Mier JW. Interleukin-6 (IL-6) as an anti-inflammatory cytokine: induction of circulating IL-1 receptor antagonist and soluble tumor necrosis factor receptor p55. Blood (1994) 83:113–8. 8274730

[72] Lin TW Cardenas L Glaser DL Soslowsky LJ. Tendon healing in interleukin-4 and interleukin-6 knockout mice. J Biomech. (2006) 39:61–9. 10.1016/j.jbiomech.2004.11.009 16271588

[73] Andersen MB Pingel J Kjaer M Langberg H. Interleukin-6: a growth factor stimulating collagen synthesis in human tendon. J Appl Physiol. (2011) 110:1549–54. 10.1152/japplphysiol.00037.2010 21350025

[74] Zhang FJ Luo W Lei GH. Role of HIF-1α and HIF-2α in osteoarthritis. Joint Bone Spine (2015) 82:144–7. 10.1016/j.jbspin.2014.10.003 25553838

[75] Murphy CL Thoms BL Vaghjiani RJ Lafont JE. HIF-mediated articular chondrocyte function: prospects for cartilage repair. Arthritis Res Ther. (2009) 11:213. 10.1186/ar2574 19232075 PMC2688225

[76] Bouaziz W Sigaux J Modrowski D Devignes C-S Funck-Brentano T Richette P et al Interaction of HIF1 alpha and beta-catenin inhibits matrix metalloproteinase 13 expression and prevents cartilage damage in mice. Proc Natl Acad Sci USA. (2016) 113:5453–8. 10.1073/pnas.1514854113 27122313 PMC4868441

[77] Risbud MV Guttapalli A Stokes DG Hawkins D Danielson KG Schaer TP et al Nucleus pulposus cells express HIF-1α under normoxic culture conditions: a metabolic adaptation to the intervertebral disc microenvironment. J Cell Biochem. (2006) 98:152–9. 10.1002/jcb.20765 16408279

[78] Schipani E Ryan HE Didrickson S Kobayashi T Knight M Johnson RS. Hypoxia in cartilage: HIF-1α is essential for chondrocyte growth arrest and survival. Genes Dev. (2001) 15:2865–76. 10.1101/gad.934301 11691837 PMC312800

[79] Duval E Baugé C Andriamanalijaona R Bénateau H Leclercq S Dutoit S et al. Molecular mechanism of hypoxia-induced chondrogenesis and its application in *in vivo* cartilage tissue engineering. Biomaterials (2012) 33:6042–51. 10.1016/j.biomaterials.2012.04.061 22677190

